# Muslim women’s experiences of maternity services in the UK: qualitative systematic review and thematic synthesis

**DOI:** 10.1186/s12884-020-2811-8

**Published:** 2020-02-18

**Authors:** Tasneema Firdous, Zoe Darwin, Shaima M. Hassan

**Affiliations:** 1 School of Healthcare, University of Leeds, Leeds, LS2 9JT UK; 2 Leeds Teaching Hospitals NHS Trust, Leeds, UK; 3 Institute of Population Health Sciences, University of Liverpool, Liverpool, UK

**Keywords:** Muslim women, Religion, Cultural competency, Maternity care, Systematic review

## Abstract

**Background:**

This review aimed to identify and synthesise evidence of Muslim women’s experiences of maternity services in the UK. A systematic review and thematic synthesis of qualitative evidence, unrestricted by type of publication was conducted. Muslim women who had accessed maternity services in the UK, regardless of obstetric or medical history were included.

**Method:**

Databases were searched from 2001 to 2019 and screened for inclusion using pre-determined criteria. The Critical Appraisal Skills Programme Qualitative Research Checklist was used to assess study quality and findings were synthesised using thematic synthesis, as described by Thomas and Harden.

**Results:**

Six studies were included. The following five themes were identified: *Islamic practices and Individualised care; Talk, Teach and Translate; Injustice, Inequity and Intolerance; If Allah wills*; and, *‘It’s not all that bad’*. Synthesis highlighted the significance of Islam in shaping many of the women’s decision-making relating to antenatal screening and medication, which was contrasted with healthcare professionals’ limited awareness of the importance of Islam for motherhood. The majority of women experienced poor maternity care which at times indicated stereotypical and discriminatory behaviour.

**Conclusions:**

Education for healthcare professionals is warranted, to enhance the quality and cultural competency in providing appropriate care that acknowledges and meets Muslim women’s needs.

## Background

Women’s social, cultural and economic circumstances can affect the choices made in pregnancy, as well as their experiences and perinatal outcomes [1, 2]. Research suggest that religion has a profound influence on women’s choices, such as timing of when to have children, size of family, decision-making on contraceptives, fertility treatments and other reproductive issues [3]. It has become increasingly important to understand the impact of faith and major spiritual events in pregnancy to ensure a woman-centred approach of care [4].

The UK has a growing multi-faith society. Muslims living in the UK are not a homogenous entity but rather, a “community of communities”, including historically, culturally, ethnically and linguistically diverse groups; some are immigrants and others are native-born [5]. Nonetheless, having a common Islamic culture, beliefs and values is recognised to connect Muslim communities, giving them some shared values with regards to health beliefs and practice; health risks; family dynamics and decision-making processes [6]. Therefore, separating Islam and culture can prove difficult, if not impossible - hence, this review focuses on Muslim women, accepting Islam as a unifying set of beliefs and practices which may be relevant to their understandings and experiences of motherhood.

Although Christianity is still the largest religion of the UK population, Muslims now occupy the second largest growing religion in the UK [7]. Healthcare professionals (HCPs) who have not yet cared for Muslims are likely to so do in the coming future. In recent years, the population of Muslims in the United Kingdom (UK) has increased from 3% in the 2001 census, to 4.8% in the 2011 census [7]. Muslims are ethnically diverse with 68% being Asian e.g. Pakistani, Indian, Bengali and 32% non-Asian; together, Muslims form one third of the Black and Minority Ethnic (BME) group in the UK [8]. Despite this, there remains little research concerning UK Muslim women’s experiences, with the majority of evidence concerning Muslim women’s experiences during pregnancy/childbearing in non-Muslim countries having been conducted in North America and Australia [9–11]. Application of findings, particularly experiences of maternity services relating to religion and culture, are likely to vary with different maternity systems and sociocultural differences.

Maternity Action, the UK’s leading charity concerned with tackling inequality in maternity, has previously argued that many UK Muslim women do not receive good quality care and basic needs are unmet [12]. Black and Minority Ethnic (BME) groups in the UK are known to have poorer experiences in maternity care, as well as adverse perinatal outcomes [13–16]. Current UK statistics also show that in pregnancy, Black women are five more times likely to die and Asian women are two times more likely to die in comparison to white women [17]. These health inequalities appear partly attributable to the provision of health [18] and the failure to provide appropriate care to BME groups in the UK [13, 15, 19]. Statistics tend to be reported by ethnicity rather than religion, however, within Muslim groups, there is similarly evidence of poor communication with HCPs, stereotyping and cultural misassumptions held by HCPs, and lack of culturally sensitive care [13, 20]. These experiences present barriers to women making informed decisions during their care, and hinder the delivery of appropriate care that meets women’s specific needs [20].

Sheikh [21] suggests the need for HCPs working in maternity services to understand Muslim women’s needs and the factors that influence their health decision-making, in order to better meet their needs. The review aims to identify and synthesise evidence of Muslim women’s experiences of maternity services in the UK.

## Methods

Reporting followed ENTREQ guidelines [22] and further details relating to the methods are available on request from the lead author, who conducted this systematic review as a final year dissertation in her pre-registration Midwifery programme.

### Search methods for identification of studies and screening for inclusion

The review question was developed using the PEOS framework where the population (P) was Muslim women in the UK, the exposure (E) was maternity services or care, the outcome (O) was experiences and the study design (S) was qualitative research. The term Muslim is ambiguous in the health literature and is often combined with ethnic group identity. Both religion and ethnicity are relevant to understanding people’s experiences of healthcare. The focus here is on religion, consistent with research finding that Muslims in the UK themselves consider religion to be an essential characteristic of their identity [21].

As shown in Table 1, terms were developed for these concepts; these were combined using Boolean terms “OR” within columns and “AND” across columns. Searching was conducted in March 2018 and updated in March 2019. With scoping work having identified that much of the literature may not be published in peer-reviewed journals, searches of electronic databases were complimented by other strategies, including reference checking, citation tracking, searching relevant websites and contacting authors to help identify grey literature.

**Table 1:** Search terms

Population	Population	Population	Exposure	Outcomes / Study Design
Muslim Islam*	Wom?n* Pregnan* Patient* Mother* Childbearing	UK United Kingdom England Scotland Wales Ireland	Maternity adj2 service* Maternity care NHS Perinatal Antenatal Postnatal *birth	Experience* Attitude* Perception* Perspective* View* Opinion* Qualitative Focus group* Interview*

Titles and abstracts were independently screened for inclusion by two reviewers (TF and SH) using pre-defined eligibility criteria (see Table 2). Those that met the criteria or did not contain adequate information to decide based on title and abstract were obtained in full and assessed by the team to determine inclusion.

**Table 2:** Eligibility criteria

	Inclusion	Exclusion
Participants	Muslim women, regardless of ethnicity, migration status, or country of birth – provided that Muslim is indicated somewhere in the text (e.g. relevant words such as Islam, Muslim, Mosque).	Non-Muslim women or mixed sample where it is not possible to disaggregate the findings according to Muslim/non-Muslim
Exposure	Maternity services in the UK	
Outcomes	Women’s experiences, views or accounts of contact with maternity services and healthcare professionals within these services, or of pregnancy / childbearing / motherhood and containing a focus on maternity services	
Study design	Qualitative research regardless of type (e.g. interpretive descriptive, phenomenology, grounded theory) or mixed-methods research containing qualitative component	Quantitative research or quantitative components of mixed-methods research; surveys that do not provide analysis of open-ended text comments
Language	Written in English language	
Publication	Any; including primary studies presented in peer-reviewed journal publications, research reports, doctoral theses	
Time period	2001–2019	

### Quality appraisal

One reviewer led (TF) quality appraisal using the Critical Appraisal Skills Programme (CASP) Qualitative Research Checklist (CASP, 2018), with assessments checked by a second reviewer (SH) and agreement reached through discussion.

### Data extraction and thematic synthesis

Different synthesis methods are available to bring together qualitative research and these vary in the extent to which they seek to summarise or further interpret the existing studies [23]. Thematic synthesis was conducted here, following the approach developed by Thomas and Harden [24]. All text in the results or findings sections of the included studies were treated as data; this therefore included participant quotations as well as interpretations by the authors of the included studies. The lead reviewer (TF) extracted the data into tables and coded each line of text according to its meaning and content. As described by Thomas and Harden [24], the codes were then organised into descriptive themes, some of which were comparable to the original findings of the included studies. The descriptive themes were then developed further into analytical themes, which developed the analysis beyond the original studies. Although conceptually similar to the third order interpretations produced using meta-ethnography, the analytical themes produced by thematic synthesis may be considered more appropriate for research that seeks to inform practice and policy [24] and thematic synthesis was therefore chosen as having good fit with the review question, specifically its applied focus on maternity services. All authors interrogated the evolving analytical themes to discuss alternative interpretations, consider their own influences (including different religious, cultural and disciplinary backgrounds), and agree a final version of the synthesis. Verbatim participants’ quotations under pseudonyms were included.

## Results

### Results of the search

**Fig. 1:**
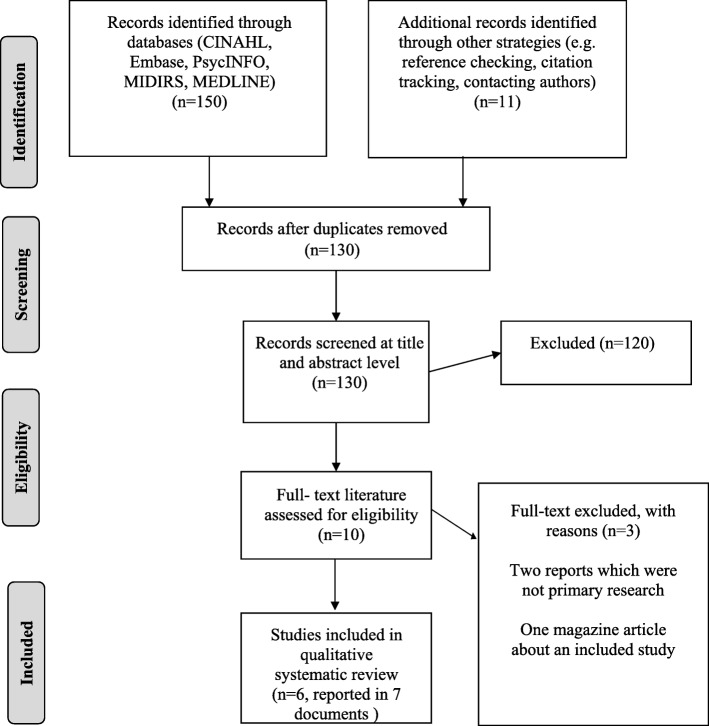
PRISMA flow diagram

As shown in Fig. 1, searching via electronic databases and additional strategies identified 130 unique records. Ten of these were obtained in full and seven (reporting six studies) met criteria for inclusion. The remaining three included two reports that did not present primary research and a magazine article that drew on the findings of one of the included studies.

### Characteristics of included studies

The six included primary research studies were reported in two journal articles, three doctoral theses (one of which underpinned one of the journal articles), one research report (conducted by a voluntary sector organisation) and one book chapter. The study characteristics are shown in Table 3.

**Table 3:** Characteristics of included studies

Study ID; Aim; Design/ Theoretical perspective	Eligibility criteria	Recruitment	Sample characteristics	Data collection and Data analysis
Study 1: Ali & Burchett, 2004 [25] To raise awareness amongst HCPs and to challenge the racial and religious stereotypes that affect Muslim women’s birth experiences. Interpretive descriptive – theoretical approach unclear	Inclusion: -Given birth within 3 years -Born and raised in UK and those born/raised overseas -Born Muslim or converted to Islam -Regardless of the women wearing the hijab (head scarf) -Regardless of income and ability to speak English	Sampling via the project’s advisory group Recruited across regions in Central and Northern England	*n* = 43 women from variety of backgrounds including Iraqi, Pakistani, Bangladeshi, European, Indian, Somali and African *n* = 22 men (partners) *n* = 8 HCPs	5 focus groups with Muslim women; Questionnaires with Muslim men; Telephone interviews with HCPs Focus group – framework analysis Open-ended question in questionnaire – content analysis HCPs telephone interviews - both framework and content analysis
Study 2: Hassan, 2017 [26]; Hassan et al., 2019 [27] To investigate Muslim women’s motherhood journey and explore the factors that influenced their health needs and decision making when engaging with services. Interpretive descriptive	Inclusion: Phase 1 and 2: First time Muslim pregnant women aged≥18 engaging with maternity services and Muslim mothers who had experienced childbirth in past 3 years. All living and received maternity care in Merseyside, England All English speaking Phase 3: HCPs from a large maternity service in Merseyside	Phase 1 and 2: Purposive sampling from the Muslim community and mailing group (used and created by a number of local Muslim women). Phase 3: Snowball sampling	Phase 1: *n* = 8 pregnant women Phase 2: *n* = 24 mothers (Varying in ethnicity, education, occupation, marital status etc.). Phase 3: *n* = 12 HCPs	Phase 1: 24 one-to-one longitudinal semi-structured interviews Phase 2: 5 focus groups Phase 3: 12 one-to-one interviews Thematic analysis
Study 3: Alshawish et al., 2013 [28] To investigate the access and use of maternity health services, as well as the barriers and facilitators for Palestinian women. Pragmatic approach, as reported by authors	Inclusion: -Aged ≥18 -Palestinian Muslim -Living in the UK -Had children in one of the Arabic schools in Manchester, England	Purposive sampling is implied and snowball technique; invited through the Arabic school and local mosque	*n* = 22 Palestinian women living in different areas of Manchester	Face-to-face, semi-structured interviews. Framework analysis
Study 4: Ellis, 2000 [29] To explore the experience of second generation South Asian Muslim women and to highlight the issues of midwifery practice for this group. Ethnography	Inclusion: -Primiparous -Aged 18–35 -Low risk pregnancy -Second generation and educated in the UK -South Asian Muslim	Purposive sampling is implied	*n* = 10; no information on ethnic background or other demographics	Semi-structured interviews 1 week after birth. Non-participant observation during labour. Review of birth-plan. Thematic analysis is implied
Study 5: Bawadi, 2009 [30] To gain insight into the lived experiences of migrant Arab Muslim women during their experiences of childbirth in the UK. Phenomenology - Heideggerian hermeneutic phenomenology	Inclusion: -Pregnant women living in and receiving maternity care in the UK -Aged ≥18 -Migrants in the UK in the past 10 years from Arab countries	Purposive sampling and snowball sampling from Islamic centre, Arabic schools and Muslim women’s societies.	*n* = 8; varying ages, parity and reason for migration	22 longitudinal semi-structured interviews carried out in the antenatal, perinatal and postnatal period. Thematic analysis and reports using an adapted version of Interpretive Phenomenological Analysis (IPA) together with hermeneutic principles.
Study 6: Bharj, 2007 [31] To explore Pakistani Muslim women’s experiences of labour and maternity services to enable the development of responsive and sensitive midwifery care and knowledge. Interpretive ethnographic	Inclusion: -Pakistani Muslim women with no history of either medical or obstetric complexities -Women identified as having healthy babies - Women were excluded if they had experienced previous obstetric care or were multigravida	Recruited from 3 cities in Northern England using the snowballing approach. Purposive sampling and convenience sampling from 9 antenatal classes	*n* = 27, including 13 primigravidae Pakistani Muslim women, 5 midwives and 9 interpreters	Semi-structured interviews and 3 participant observations during labour. Content analysis (thematic framework approach)

### Quality appraisal

The CASP [32] appraisals are summarised in Table 4. All six studies had clear objective aims which integrated with their chosen methodologies. The theoretical approaches were clearly stated in studies 2 and 6.

**Table 4:** Summary of critical appraisal using the qualitative CASP checklist

Study ID	1	2	3	4	5	6
1) Was there a clear statement of the aims of the research?	Yes	Yes	Yes	Yes	Yes	Yes
2) Is a qualitative methodology appropriate?	Yes	Yes	Yes	Yes	Yes	Yes
3) Was the research design appropriate to address the aim of the research?	Unclear	Yes	Unclear	Unclear	Yes	Yes
4) Was the recruitment strategy appropriate to the aims of the research?	Unclear	Yes	Unclear	Unclear	Yes	Yes
5) Was the data collected in a way that it addressed the research issue?	Unclear	Yes	Yes	Unclear	Yes	Yes
6) Has the relationship between the researcher and participants been adequately considered?	No	Yes	Yes	Yes	Yes	Yes
7) Have ethical issues been taken into consideration?	Yes	Yes	Yes	Yes	Yes	Yes
8) Was the data analysis sufficiently rigorous?	No	Yes	Yes	Yes	Yes	Yes
9) Is there a clear statement of findings?	Unclear	Yes	Yes	Yes	Yes	Yes
10) Is the research valuable?	Yes	Yes	Yes	Yes	Yes	Yes

All the studies used a purposive sampling method with studies 2,3 and 6 also using snowball sampling. Only studies 2,5 and 6 clearly justified the sampling choices. Studies 2, 5 and 6 provided detail with regards to discussion of data saturation and participant withdrawal. Ethical considerations were evident in studies 2,3,5 and 6.

Due to the lack of current research surrounding Muslim women specifically, grey literature was also included. It is therefore unsurprising that the included studies were of variable quality, due to not having all gone through the peer review process. Overall, it was evident that the research reported in the three doctoral theses (studies 2,5,6) were of higher quality and showed high levels of transparency. It is recognised however that theses face fewer limitations regarding reporting length of restrictions found in journal publications. Study 1 presented some methodological concerns but was nonetheless included in the synthesis, which is acceptable with the method of synthesis used [24].

### Thematic synthesis

Thematic synthesis identified the following five analytical themes: (1) *Islamic practices and Individualised care;* (2) *Talk, Teach and Translate;* (3) *Injustice, Inequity and Intolerance;* (4) *If Allah wills*; and, (5) *‘It’s not all that bad’*.

#### Theme 1: Islamic practices and individualised care

This theme investigates the common concepts of privacy, modesty, antenatal screening, religious practices (such as *Adhan [Islamic call to prayer], Tahneek [Rubbing of something sweet (*e.g. *a date) on a new-borns tongue], fasting the month of Ramadhan* [*ninth month of the Islamic calendar, which requires one to fast]*), dietary requirements and the presence of males. There was a lack of understanding and awareness regarding Muslim women’s decision-making process (Studies 1–6). However, these concepts impacted Muslim women’s choice during maternity, for example the presence of men in antenatal classes and within hospital wards impacted women’s engagement with antenatal classes and breastfeeding.

*“ … I don’t like to attend these classes, because it is uncomfortable … as it is women mixed with men. Where is my privacy with these men … ”* Zahra [*pseudonym*] (**participant quotation, Study 5, pg.223***)*.

Women in study 2 lacked choice regarding availability of appropriate meals that met dietary requirements regarding the women’s needs of having *Halal* [denoting or relating to meat prepared as prescribed by Muslim law] food; they also lacked detailed information regarding medication and whether it was free from animal-based products. For some women in the included studies, choices in birth plans were not appropriately acknowledged by HCPs.

*“I forgot to mention some Islamic practices … when I came to tell her that I wanted to add points to my birth plan she looked annoyed and said “we have done this last week … ”* Sahar [*pseudonym*] (**participant quotation, Study 2, pg.131**).

Religious practices such as *Adhan, Tahneek* and fasting were not expressed by women due to potential misperceptions or misunderstanding from the HCPs (studies 2, 5 and 6). The majority of women did not discuss screening options with HCPs but decided to decline screening tests such as Down syndrome screening (studies 2, 3, 5 and 6).

*“ … I was 25 weeks pregnant and I decided not to have an abortion because of religious issues, we believe in destiny and fate”* Participant number 5 (**participant quotation, Study 3, pg.573**)

Lack of sensitive and emotional care was most evident in study 5, which investigated migrant women’s experiences. Women made comparisons between the quality of care in their country in contrast to the NHS. Concepts of vulnerability and lack of family support were prominent in study 5 and had a great impact on women’s experiences within the NHS.

*“ … will they cover me as they do in Jordan? This is an important issue for me...”* Rahma [*pseudonym*] (**participant quotation, Study 5, pg.131**)

All the women in the included studies appreciated continuity of care as this enabled trusting relationships to form. However, women stated a preference for Muslim HCPs, noting they would already have an understanding of these practices, making women more likely to express their wishes (study 2).

*“I think with a Muslim midwife you would feel more comfortable telling her things … you can easily tell a Muslim midwife that you want your child to hear Allah (God) and she would completely understand.”* Noor [*pseudonym*] (**participant quotation, Study 2, pg.136**)

#### Theme 2: talk, teach and translate

Language barriers, educational resources, informed consent and the use of translators were concepts running thorough all six studies. Women expressed the need for religious-related information from HCPs or an understanding of these requirements; many women had turned to friends and other sources due to lack of awareness amongst HCPs.

*“I bought magazines and read about birth of baby … When I used to ask my midwife for information, she just didn’t have time to discuss other things”* Maheera [*pseudonym*] (**participant quotation, Study 6, pg. 132**)

Those that did receive leaflets and were informed of the services reported finding them helpful.

*“During my first pregnancy they gave me information. I went every Thursday with my husband. It was useful.”* Amina [*pseudonym*] (**participant quotation, Study 5, pg.224**)

However, not all women were able to speak or read English, therefore the leaflets were of little use and there was risk of compromised ability for informed choice. The need for understanding medical terminology was evident, as well as the importance of translating services.

*“ … I could not understand everything they said. I told my husband to translate everything for me but he did not. He was hiding the truth and trying to comfort me”* Fatima [*pseudonym*] (**participant quotation, Study 5, pg.193**)

“ *… women who do not speak English that is an issue in our Asian culture you know interpreting is an issue”* Khatiza [*pseudonym*] (**participant quotation, Study 6, pg. 127**)

Informed choice and consent were major factors in women’s experiences. Women appreciated when their wishes were acknowledged or when they were informed of changing situations.

*“She prepared me beforehand [for the fact that a] male doctor might examine me, and if this happened, I should not be angry because she gave me the background about the conditions”.* Kawther [*pseudonym*] (**participant quotation, Study 5, pg.137**)

#### Theme 3: injustice, inequity and intolerance

This theme identifies women’s experiences in relation to stereotype, discrimination and prejudice (studies 1, 2, 4, 5 and 6). Study 4 framed the findings in the context of institutionalised racism with respect to organisational power and hierarchies between HCPs and social classes. Across all six studies, women themselves formed a link between insensitive care and racism. Women felt that their clothing e.g. veil, *Hijab [a veil used as a head covering] /Abaiya [A full length garment]* clearly identified them as Muslims and therefore made them more prone to discrimination, including from HCPs.

*“The manner in which she talked to me was very bad … It was clearly because I am a Muslim and wear a veil … ”* Rahma [*pseudonym*] (**participant quotation, Study 5, pg.197**)

#### Theme 4: if Allah wills

In all six studies, women reported that their experiences were influenced by their spirituality and faith; this was more pronounced in studies 2, 5 and 6.

*“It is such a spiritual journey … motherhood journey would make you gain some Iman (faith)”* Gp4; P1 (**participant quotation, Study 2, pg.109**)

*“I was reading “Quran [An Islamic sacred book of god, revealed to the prophet] Shareef’ and I got the internal peace and internal strength to cope with the pain.”* Lateefa [*pseudonym*] (**participant quotation, Study 6, pg.169**)

In studies 2 and 5 some women discussed how spirituality helped their mental health. They used the Quran and calling on God for support during the time of struggle.

*“Without my faith … I would probably go through depression”* Hanan [*pseudonym*] (**participant quotation, Study 2, pg.96**)

This also influenced some women’s choice on declining things such as Down syndrome screening, whereby they identified a state of acceptance regarding the child’s health, i.e. ‘if Allah wills’ (study 2).

#### Theme 5: It’s not all that bad’

The majority of the participants reported poor experiences however positive experiences were also evident in studies; the exception being study 4.

*“I never felt discriminated against on the grounds of my race. On the contrary, I felt that they respected our religion. In the midwife’s first home visit she said “Al-salaam alykom” in Arabic instead of “hello”.”* Amal [*pseudonym*] (**participant quotation, Study 5, pg.197**)

*“Midwives respected not only women’s faith and culture but their privacy, and as a result woman stated that they had a sense of control over their body and their care.”* (**Author quotation, Study 6 pg. 165**).

It appeared that positive experiences were related to the midwife having an understanding of Islam and having an individualised outlook on care.

## Discussion

This systematic review identified five themes surrounding Muslim Women’s experiences of maternity services in the UK, which captured influences on women’s decision-making, HCPs’ awareness of Islam and religious practices, women’s experience of poor maternity care, experiences of negative encounters and positive examples of care. These demonstrate the importance of understanding the impact of religion – Islam - within maternity services. It is evident that although women’s ethnicities varied, their common ground of Islam and spirituality is at the pinnacle of their worldly priorities and way of living. Nonetheless, women require individualised care and Muslim women should not be treated as a homogenous group.

Although Muslim women expressed some positive experiences while engaging with maternity services, there were still common negative experiences encounter by the majority of women. HCPs came across as insensitive to Muslim women’s needs due to a lack of understanding of religious values and practices. This impacted Muslim women’s confidence in discussing their specific needs, making them more reluctant to form or discuss their birth plans with HCPs. Women expressed concerns that HCPs may not appropriately acknowledge their needs discussed or highlighted on their birth plan.

Religious beliefs have been shown to affect choices made in relation to medication and antenatal screening [33]. The majority of the women’s comments were in relation to midwives specifically. It is therefore important to educate HCPs including midwives to increase awareness of Islam, the beliefs and birth customs, to develop culturally competent care and facilitate woman-centred care when caring for Muslim women [26, 27]. Further challenges at the system level are improving verbal and non-verbal communication, increasing the availability of interpreters, and emphasising the importance of continuity of care [31]. This would have a positive impact on women’s health outcomes and contribute towards improving experiences for BME women, more widely.

### Individualised care and maternity services

Individualised woman-centred care features in international policy relating to midwifery and in the UK has featured consistently, including in the most recent National Maternity Review [34]. Midwifery care presents numerous opportunities for being individualised; one example being birth plans. Study found that birth plans can improve the skilled care provided during pregnancy and was found to improve women’s engagement within maternal health services [35]. However, some women in the included studies felt that birth plans were not assigned importance and that their stated preferences were not appropriately acknowledged.

The majority of the women declined prenatal screening; this coheres with wider literature, which reports lower frequencies in genetic screening e.g. for Down’s syndrome, especially within Arab women [36]. Despite the introduction of new non-invasive prenatal screening tests (NIPT), the aspect of abortion is strongly prohibited in Islam [37] and may therefore not substantially change uptake. Furthermore, prominent in the synthesis was concerns relating to the presence of males; a finding that is consistent across the international literature and identified as the main reason for Muslim women being less likely to attending antenatal classes, due to the presence of other males [38, 39].

Continuity of care has shown to maintain women-centred care, reduce the risk of complications, improve documentation and also improves patient satisfaction [40]. Where women in this review had received continuity of care, it was valued highly, and appeared to facilitate individualised care; however, continuity of care itself does not ensure individualised care. Mari [41], argues that woman-centred care can only be achieved by understanding women, their backgrounds and religious beliefs which consequently impacts the choices made and the relationships formed.

### Communication

Poor communication is the main barrier to the effective use of maternal and child health services [19]. Availability of professional interpreters was identified as problematic in some of the studies and negatively impact women’s experiences. Some women preferred their family members to translate however this is not without concern [42]. For example, family members who interpret have been shown to have a higher incidence of incorrect translation of phrases and medical terminology, with potential to compromise patient care by increasing the risk of clinical complications [42].

Both verbal and non-verbal forms of communication are vital in portraying sensitivity and providing appropriate care [43]. Even without the required availability of interpreter services, women’s experiences indicated that non-verbal communication may not have met expected standards. Thus, both improved availability of interpreters and improved verbal and non-verbal communication are needed, to promote women-centred care.

### Discrimination and stereotypes

Bowler [44] was the first to explore how midwives use stereotyping (i.e. oversimplified generalisations about groups of people that do not account for individual differences) to make assumptions regarding the care that Asian women may need, and furthermore that inequality within BME groups may be in part influenced by prejudiced attitudes and discriminatory behaviours. These included studies provide concerning examples of where women have experienced actions as discriminatory, despite the introduction of equality and diversity policies across the NHS. Such examples could be used to inform training and the provision of future anti-discriminatory policies, which appears increasingly relevant in the current climate of growing Islamophobia [45]. Further high-quality research is required in this area.

### Spirituality

Although there is some recognition that spirituality impacts pregnancy [3, 46, 47], this remains an under-researched area. Religion is a major aspect of a Muslim women’s life and could potentially help reduce the risk of postnatal depression [48], as indicated in one of the included studies. Further research regarding Islam and perinatal mental health is warranted.

### The positives

Whilst the majority of experiences were negative in the included studies, five of the six studies contained some comments indicating positive experiences. For example, in studies 5 and 6, women expressed the value of respect and understanding of their faith and the difference it made with their relationship with their midwife and felt they had a better maternity experience because of this.

#### Strengths and limitations

There is no accepted minimum or maximum number of studies for inclusion in a qualitative synthesis [49] and the reviewers were satisfied that the included studies are suitably rich to provide a meaningful synthesis, which is relevant for building confidence in the findings of the synthesis [49]. It is notable that despite there being six studies eligible for inclusion, only two were published in a peer reviewed journal, which may raise questions concerning potential publication bias. As study 5 looked at migrant Muslim women, these enabled a platform of comparison in the differing maternity services. Despite varied quality, including three robust studies reported in doctoral dissertations, it was notable that findings across studies were largely consistent. The review’s scope could have been widened by including relevant studies that did not report religion but included women from different ethnic groups such as Pakistan, Bangladesh, or Somali, who predominately identify as Muslim. This could have led to inclusion of more studies from the peer-reviewed literature giving a wider range of experiences, however, this review specifically focused on studies that explicitly identified Muslim women, reflecting calls for more research to consider women’s religious identity. In addition, broadening the scope may have compromised the review being manageable [50].

## Conclusion

The review clearly indicates that further improvements are required to provide culturally sensitive, competent and individualised care. Further education is needed to equip HCPs with greater understanding of the potential influence of Islam on women’s decision-making processes and further training for improved communication, supported by interpreters where required. Women’s reports indicate concerning comments relating to discrimination that need to be challenged through anti-discrimination policies. There is an increase in the visibility of a Muslim workforce which may further support these changes.

## Data Availability

Not applicable.

## Abbreviations

BME: Black and Minority Ethnics
CASP: Critical Appraisal Skills Programme
HCP: Health Care Professionals
NIPT: Non-invasive prenatal screening tests

## References

[1] Robab LR (2015). Socio-cultural believes values and traditions regarding Women’s preferred mode of birth in the north of Iran. Int J Community Nurs Midwifery.

[2] Larson PC (2007). Poverty during pregnancy; its effects on child health outcomes. Paediatr Child Health.

[3] Gaydos LM (2010). An emerging field in religion and reproductive health. J Relig Health.

[4] Jesse DE (2007). The effect of faith or spirituality in pregnancy. J Holist Nurs.

[5] Rassool GH (2014). Cultural competence in caring for Muslim patients.

[6] Rassool GH (2015). Cultural competence in nursing Muslim patients. Nurs Times.

[7] Office for National Statistics (ONS) (2012). Religion in England and Wales 2011.

[8] Muslim Council of Britain MCB (2015). British Muslim Number: a demographic, socio-economic and health profile of Muslim in Britain drawing on the 2011 census.

[9] Hasnain M (2011). Patient- centred care for Muslim women; provider and patient perspectives. J Women's Health.

[10] Gustafson DL, Reitmanova S (2008). “They Can’t Understand It”: Maternity Health and Care Needs of Immigrant Muslim Women in St. John’s, Newfoundland. Matern Child Health J.

[11] Tsianakas V, Liamputtong P (2002). What women from an Islamic background in Australia say about care in pregnancy and prenatal testing?. Midwifery.

[12] Pollock L (2005). Experiences of Maternity Services: Muslim Women’s Perspectives.

[13] Lewis G. Saving Mothers’ Lives: reviewing maternal deaths to make motherhood safer – 2003–2005. The Seventh Report of Confidential Enquiries into Maternal Deaths in the United Kingdom. The Confidential Enquiry into Maternal and Child Health (CEMACH) London. CEMACH. 2007; Available from: http://www.publichealth.hscni.net/sites/default/files/Saving%20Mothers%27%20Lives%202003-05%20.pdf, [Online]. [Accessed 9th March 2018].

[14] Patrick ET, Bryan Y (2005). Research strategies for optimizing pregnancy outcomes in minority populations. Am J Obstet Gynaecol.

[15] Henderson J (2013). Experiencing maternity care: the care received and perceptions of women from different ethnic groups. BMC Pregnancy Childbirth.

[16] Puthussery S (2016). Perinatal outcomes among migrant mothers in the United Kingdom: is it a matter of biology, behaviour, policy, social determinants or access to health care?. Best Pract Res Clin Obstet Gynaecol.

[17] Knight (2018). MBRACE- UK, Saving Lives, Improving Mothers’ Care.

[18] The Marmot Review (2012). Fair Society, Healthy Lives. Strategic Review of Health Inequalities in England post-2010.

[19] Straus L (2009). Somali women’s experience of childbirth in the UK: perspectives from Somali health workers. Midwifery.

[20] McFadden A (2013). Does cultural context make a difference to women’s experiences of maternity care? A qualitative study comparing the perspectives of breast-feeding women of Bangladeshi origin and health practitioners. Health Expect.

[21] Sheikh A (2007). Should Muslims have a faith-based health service?. Br J Midwifery.

[22] Tong A (2012). Enhancing transparency in reporting the synthesis of qualitative research: ENTREQ. BMC Med Res Methodol.

[23] Barnett-Page E, Thomas J (2009). Methods for the synthesis of qualitative research: a critical review. BMC Med Res Methodol.

[24] Thomas J, Harden A (2008). Methods for thematic synthesis of qualitative research in systematic reviews. BMC Med Res Methodol.

[25] Ali N, Burchett H. Experiences of maternity services: Muslim women's perspectives. Maternity Alliance. 2004; Available from: https://www.maternityaction.org.uk/wp-content/uploads/2013/09/muslimwomensexperiencesofmaternityservices.pdf. [Online]. [Accessed 10th March 2018].

[26] Hassan SM. A qualitative study exploring British Muslim women’s experiences of motherhood while engaging with NHS maternity services: Doctoral Thesis, Liverpool John Moores University; 2017. Available from http://researchonline.ljmu.ac.uk/7412/7/2017ShaimaHassanPhD.pdf, [Online]. [Accessed 10th March 2018]

[27] Hassan MS, Leavey C, Rooney JS (2019). Exploring English speaking Muslim women’s first-time maternity experiences: a qualitative longitudinal interview study. BMC Pregnancy Childbirth J.

[28] Alshawish E, Wibberley C, Marsden J, Yeowell G (2013). Investigating access to and use of maternity health-care services in the UK by Palestinian women. Br J Midwifery.

[29] Ellis N, Kirkham M (2000). ‘Birth Experiences of South Asian Muslim Women: Marginalised Choice within the Maternity Services. Informed choice in maternity care.

[30] Bawadi H. Migrant Arab Muslim Women’s experiences of childbirth in the UK: Doctoral Thesis, De Montfort University; 2009. Available from: https://www.dora.dmu.ac.uk/bitstream/handle/2086/3039/Thesis.pdf?sequence=1&isAllowed=y, [Online]. [Accessed 10th March 2018]

[31] Bharj KK. Pakistani Muslim women birthing in northern England: exploration of experiences and context: Doctoral Thesis, Sheffield Hallam University; 2007. Available from: http://shura.shu.ac.uk/20627/1/10701274.pdf. [Online]. [Accessed 10th August 2018]

[32] Critical Appraisal Skills Programme (CASP) (2018). CASP (Qualitative Research) Checklist.

[33] Gitsels-van der Wal JK (2014). The role of religion in decision-making on antenatal screening of congenital anomalies: a qualitative study amongst Muslim Turkish origin immigrants. Midwifery.

[34] Births B (2017). National Maternity Review, improving outcomes of maternity services in England; A five year forward view for maternity care.

[35] Magoma M (2013). The effectiveness of birth plans in increasing use of skilled care at delivery and postnatal care in rural Tanzania: a cluster randomised trial. Tropical Med Int Health.

[36] Rassin M (2009). Cultural differences in child delivery: comparisons between Jewish and Arab women in Israel. Int Nurs Rev.

[37] Haider H (2015). Non-invasive prenatal testing: implications for Muslim communities. AJOB Empirical Bioethics.

[38] Hammad A, et al. Guide to Arab culture: Health care delivery to the Arab American community. ACCESS Community Health and Research Centre, USA. 1999. https://cdn.vanderbilt.edu/vu-my/wp-content/uploads/sites/666/2013/04/14102318/Guide-to-Arab-Culture-Health-care-delivery-to-the-Arab-American-Community.pdf.

[39] Raleigh VS (2010). Ethnic and social inequalities in women’s experience of maternity care in England: results of a national survey. J R Soc Med.

[40] Royal College of Midwives (RCM) (2014). High Quality Midwifery Care.

[41] Mari P. Women centred care? An exploration of professional care in midwifery practice: Doctoral Thesis, University of Huddersfield; 2009. Available from: http://eprints.hud.ac.uk/id/eprint/5764/1/PhD_THESIS_MARCH_2009.pdf, [Online]. [Accessed 13th June 2018]

[42] Sainath N (2011). Twisted Translation: Using Friends and Family Members As Medical Interpreters.

[43] Mast MS (2007). On the importance of nonverbal communication in the physician-patient interaction. Patient Educ Couns.

[44] Bowler I (1993). ‘They’re not the same as us’: midwives’ stereotypes of south Asian descent maternity patients. Sociol Health Illn.

[45] Runnymede (2017). Islamophobia; Still a challenge for us all; A 20th anniversary report.

[46] Callister C (2010). L and Khalaf, I. spirituality in childbearing women. J Perinat Educ.

[47] Jesse DE, Alligood MR (2002). Holistic obstetrical problem evaluation (HOPE): testing a theory to predict birth outcomes in a group of women from Appalachia. Healthc Women Int.

[48] Zaidi A (2017). Perinatal mental health and Islam. Br J Midwifery.

[49] Lewin S, Glenton C, Munthe-Kaas H, Carlsen B, Colvin CJ, Gülmezoglu M (2015). Using qualitative evidence in decision making for health and social interventions: an approach to assess confidence in findings from qualitative evidence syntheses (GRADE-CERQual). PLoS Med.

[50] Soilemezi D, Linceviciute S (2018). Synthesizing qualitative research: reflections and lessons learnt by two new reviewers. Int J Qual Methods.

